# Genotypic effects of *APOE*-ε4 on resting-state connectivity in cognitively intact individuals support functional brain compensation

**DOI:** 10.1093/cercor/bhac239

**Published:** 2022-06-27

**Authors:** Raffaele Cacciaglia, Grégory Operto, Carles Falcón, José Maria González de Echavarri-Gómez, Gonzalo Sánchez-Benavides, Anna Brugulat-Serrat, Marta Milà-Alomà, Kaj Blennow, Henrik Zetterberg, José Luis Molinuevo, Marc Suárez-Calvet, Juan Domingo Gispert, for the ALFA study, Müge Akinci, Annabella Beteta, Alba Cañas, Irene Cumplido, Carme Deulofeu, Ruth Dominguez, Maria Emilio, Karine Fauria, Sherezade Fuentes, Oriol Grau-Rivera, Laura Hernandez, Gema Huesa, Jordi Huguet, Eider M Arenaza-Urquijo, Eva M Palacios, Paula Marne, Tania Menchón, Carolina Minguillon, Eleni Palpatzis, Cleofé Peña-Gómez, Albina Polo, Sandra Pradas, Blanca Rodríguez-Fernández, Aleix Sala-Vila, Gemma Salvadó, Mahnaz Shekari, Anna Soteras, Laura Stankeviciute, Marc Vilanova, Natalia Vilor-Tejedor

**Affiliations:** Barcelonaβeta Brain Research Center (BBRC), Pasqual Maragall Foundation, Wellington 30, 08005 Barcelona, Spain; Hospital del Mar Medical Research Institute (IMIM), 08005 Barcelona, Spain; Centro de Investigación Biomédica en Red de Fragilidad y Envejecimiento Saludable (CIBERFES), 28089 Madrid, Spain; Barcelonaβeta Brain Research Center (BBRC), Pasqual Maragall Foundation, Wellington 30, 08005 Barcelona, Spain; Hospital del Mar Medical Research Institute (IMIM), 08005 Barcelona, Spain; Centro de Investigación Biomédica en Red de Fragilidad y Envejecimiento Saludable (CIBERFES), 28089 Madrid, Spain; Barcelonaβeta Brain Research Center (BBRC), Pasqual Maragall Foundation, Wellington 30, 08005 Barcelona, Spain; Hospital del Mar Medical Research Institute (IMIM), 08005 Barcelona, Spain; Centro de Investigación Biomédica en Red de Bioingeniería, Biomateriales y Nanomedicina (CIBERBBN), 28089 Madrid, Spain; Barcelonaβeta Brain Research Center (BBRC), Pasqual Maragall Foundation, Wellington 30, 08005 Barcelona, Spain; Hospital del Mar Medical Research Institute (IMIM), 08005 Barcelona, Spain; Centro de Investigación Biomédica en Red de Fragilidad y Envejecimiento Saludable (CIBERFES), 28089 Madrid, Spain; Barcelonaβeta Brain Research Center (BBRC), Pasqual Maragall Foundation, Wellington 30, 08005 Barcelona, Spain; Hospital del Mar Medical Research Institute (IMIM), 08005 Barcelona, Spain; Centro de Investigación Biomédica en Red de Fragilidad y Envejecimiento Saludable (CIBERFES), 28089 Madrid, Spain; Barcelonaβeta Brain Research Center (BBRC), Pasqual Maragall Foundation, Wellington 30, 08005 Barcelona, Spain; Hospital del Mar Medical Research Institute (IMIM), 08005 Barcelona, Spain; Centro de Investigación Biomédica en Red de Fragilidad y Envejecimiento Saludable (CIBERFES), 28089 Madrid, Spain; Barcelonaβeta Brain Research Center (BBRC), Pasqual Maragall Foundation, Wellington 30, 08005 Barcelona, Spain; Hospital del Mar Medical Research Institute (IMIM), 08005 Barcelona, Spain; Centro de Investigación Biomédica en Red de Fragilidad y Envejecimiento Saludable (CIBERFES), 28089 Madrid, Spain; Universitat Pompeu Fabra, 08002 Barcelona, Spain; Department of Psychiatry and Neurochemistry, Institute of Neuroscience and Physiology, The Sahlgrenska Academy at the University of Gothenburg, 41390 Mölndal, Sweden; Clinical Neurochemistry Laboratory, Sahlgrenska University Hospital, 41390 Mölndal, Sweden; Department of Psychiatry and Neurochemistry, Institute of Neuroscience and Physiology, The Sahlgrenska Academy at the University of Gothenburg, 41390 Mölndal, Sweden; Clinical Neurochemistry Laboratory, Sahlgrenska University Hospital, 41390 Mölndal, Sweden; UK Dementia Research Institute at UCL, WC1E 6BT London, United Kingdom; Department of Neurodegenerative Disease, UCL Institute of Neurology, WC1N 3BG London, United Kingdom; Honk Kong Center for Neurodegenerative Diseases, Hong Kong, China; Barcelonaβeta Brain Research Center (BBRC), Pasqual Maragall Foundation, Wellington 30, 08005 Barcelona, Spain; Barcelonaβeta Brain Research Center (BBRC), Pasqual Maragall Foundation, Wellington 30, 08005 Barcelona, Spain; Hospital del Mar Medical Research Institute (IMIM), 08005 Barcelona, Spain; Centro de Investigación Biomédica en Red de Fragilidad y Envejecimiento Saludable (CIBERFES), 28089 Madrid, Spain; Servei de Neurologia, Hospital del Mar, Barcelona, Spain; Barcelonaβeta Brain Research Center (BBRC), Pasqual Maragall Foundation, Wellington 30, 08005 Barcelona, Spain; Hospital del Mar Medical Research Institute (IMIM), 08005 Barcelona, Spain; Centro de Investigación Biomédica en Red de Bioingeniería, Biomateriales y Nanomedicina (CIBERBBN), 28089 Madrid, Spain; Universitat Pompeu Fabra, 08002 Barcelona, Spain

**Keywords:** Alzheimer’s disease, APOE-ε4, resting-state connectivity, compensation

## Abstract

The investigation of resting-state functional connectivity (rsFC) in asymptomatic individuals at genetic risk for Alzheimer’s disease (AD) enables discovering the earliest brain alterations in preclinical stages of the disease. The *APOE*-ε4 variant is the major genetic risk factor for AD, and previous studies have reported rsFC abnormalities in carriers of the ε4 allele. Yet, no study has assessed *APOE*-ε4 gene-dose effects on rsFC measures, and only a few studies included measures of cognitive performance to aid a clinical interpretation. We assessed the impact of *APOE*-ε4 on rsFC in a sample of 429 cognitively unimpaired individuals hosting a high number of ε4 homozygotes (*n* = 58), which enabled testing different models of genetic penetrance. We used independent component analysis and found a reduced rsFC as a function of the *APOE*-ε4 allelic load in the temporal default-mode and the medial temporal networks, while recessive effects were found in the extrastriate and limbic networks. Some of these results were replicated in a subsample with negative amyloid markers. Interaction with cognitive data suggests that such a network reorganization may support cognitive performance in the ε4-homozygotes. Our data indicate that *APOE*-ε4 shapes the functional architecture of the resting brain and favor the idea of a network-based functional compensation.

## Introduction

Resting-state functional connectivity (rsFC) provides access to the intrinsic organizational properties of the brain, and it has become an increasingly valued imaging biomarker for Alzheimer’s disease (AD) (van den Heuvel and Sporns 2019). Robust evidence collected over years has shown a disrupted rsFC in AD dementia patients (Badhwar et al. 2017) particularly within the default-mode network (DMN) (Greicius et al. 2004; Rombouts et al. 2005). The evidence that spatial patterns of beta-amyloid (Aβ) accumulation in the brain overlap with the DMN regions (Buckner et al. 2005; Palmqvist et al. 2017) has prompted researchers to further examine the role of DMN in AD. Hence, it has been shown that a reduced DMN connectivity correlates with disease severity (Petrella et al. 2011; Brier et al. 2012) and tracks disease progression (Damoiseaux, Prater, et al. 2012a). Moreover, there is evidence that AD patients are characterized by impaired rsFC in other brain networks, such as the executive control (Agosta et al. 2012) and the salience networks (Zhou et al. 2010).

The investigation of rsFC in asymptomatic individuals at genetic risk for AD represents a valuable tool for the identification of intermediate phenotypes, which may be informative on the earliest brain alterations in the preclinical stages of the disease (Foo et al. 2020). The *APOE* polymorphism is the most commonly studied variant in association with different AD neuroimaging markers (Fouquet et al. 2014). *APOE*-ε4 is a common yet highly penetrant variant conferring a higher risk for AD, in a gene dose-dependent manner (Corder et al. 1993; Farrer et al. 1997). Neuroimaging studies of *APOE*-ε4 in cognitively unimpaired (CU) individuals have identified dose-dependent effects on Aβ deposition (Reiman et al. 2009; Morris et al. 2010) and cerebral metabolism (Reiman et al. 2005; Protas et al. 2013). We have recently extended those previous findings of gene-dose effects to the morphological properties of the brain (Cacciaglia, Molinuevo, Sanchez-Benavides, et al. 2018a; Cacciaglia et al. 2019; Martí-Juan et al. 2021) as well as to the white matter microstructure (Operto et al. 2019), in middle-aged CU individuals. Studies investigating the impact of *APOE*-ε4 on rsFC in asymptomatic individuals have so far revealed heterogeneous findings. Most of them focused on the DMN, with both increased (Filippini et al. 2009; Fleisher et al. 2009) and decreased connectivity being reported (Machulda et al. 2011; Damoiseaux, Seeley, et al. 2012b; Patel et al. 2013). Others have reported a reduced FC in the medial temporal lobe using measures of local efficiency (Chen et al. 2015), or more conventional seed-based approaches (Heise et al. 2014), and one study reported a decreased rsFC between 2 major cognitive networks (i.e. the executive control and the salience networks) in a cross-sectional analysis, along with an increased connectivity between the DMN and the ECN when assessed longitudinally, suggesting a lack of functional segregation in *APOE*-ε4 carriers compared with noncarriers (NC; Ng et al. 2018). Importantly, however, the dose-dependent impact of *APOE*-ε4 on rsFC in CU individuals has not been yet addressed. In addition, only a few studies have concomitantly assessed cognitive performance in association with rsFC (Westlye et al. 2011; Shafer et al. 2021), to ascertain the functional implication of brain network differences in at-risk individuals. Therefore, in the present study, we aim to characterize the impact of *APOE*-ε4 in CU individuals, testing different models of genetic penetrance across multiple resting-state networks, to enable determining putative dose-dependent effects. To aid our understanding of the clinical implication of group network differences, we further investigated whether *APOE*-ε4 modulated the association between rsFC and incipient structural degeneration as well as cognitive performance in multiple domains. Finally, given that cerebral Aβ deposition has been related to disrupted rsFC in asymptomatic individuals (Sperling et al. 2009; Mormino et al. 2011; Lim et al. 2014), in the attempt of isolating gene-specific effects from those of AD pathology, we repeated all analyses in a subsample of individuals with negative Aβ cerebrospinal fluid (CSF) markers.

## Materials and methods

### Study participants

All participants were enrolled in the ALFA (ALzheimer and FAmilies) study (Clinicaltrials.gov Identifier: NCT01835717), a research platform aiming at identifying the pathophysiological alterations in preclinical AD. The ALFA cohort comprises 2743 CU individuals, with a Clinical Dementia Rate score of 0, most of them being first-order descendants of AD patients (Molinuevo et al. 2016). Subjects with a psychiatric diagnosis were excluded from the study. Additional exclusion criteria were described in detail previously (Molinuevo et al. 2016). After *APOE* genotyping, all participants homozygous for the ε4 allele as well as carriers of the ε2 allele were invited to undergo magnetic resonance imaging (MRI) scanning along with ε4-heterozygotes and NC matched for age and sex. This recruitment strategy resulted in 576 study participants, out of which 43 had to be discarded due to MRI incidental findings or poor image quality (Brugulat-Serrat et al. 2017). Of the remaining 533 participants, 104 had to be discarded due to excessive movement or intensity artifacts, leading to a final sample of 429 participants. The study was approved by the Independent Ethics Committee “Parc de Salut Mar,” Barcelona, and all participants gave written informed consent.

### APOE genotyping

Total DNA was obtained from blood cellular fraction by proteinase K digestion followed by alcohol precipitation. Samples were genotyped for 2 single-nucleotide polymorphisms, rs429358 and rs7412, determining the possible *APOE* isoforms: ε1, rs429358 (C) + rs7412 (T); ε2, rs429358 (T) + rs7412 (T); ε3, rs429358 (T) + rs7412 (C); and ε4, rs429358 (C) + rs7412 (C). Of the 429 participants, 130 were ε3/ε4 carriers, 113 were homozygous for the ε3 allele, 87 were ε2/ε3 carriers, 58 were homozygous for the ε4 allele, 36 were ε2/ε4, and 5 were homozygous for the ε2 allele. The allele frequencies did not significantly deviate from Hardy–Weinberg equilibrium (χ^2^ = 5.99, *P* = 0.20).

### Image data acquisition and preprocessing

The functional MRI (fMRI) session was performed with a 3T General Electric full body scanner (GE Discovery MR750 W) equipped with a 32-channel transmit-receiver phase array coil. One hundred twenty whole-brain resting-state blood oxygenation level-dependent (BOLD) functional volumes were acquired per subject, with the following parameters: voxel size = 3 mm^3^ isotropic, repetition time (TR) = 3,000 ms, echo time (TE) = 30 ms, matrix size = 64 × 64, flip angle = 81°.

A structural 3D high-resolution T1-weighted image was additionally collected for each participant, using a fast spoiled gradient-echo sequence with the following parameters: voxel size = 1 mm^3^ isotropic, TR = 6.16 ms, TE = 2.33 ms, inversion time = 450 ms, matrix size = 256 × 256 × 174, and flip angle = 12°. Preprocessing of the functional images was conducted in SPM12 (Wellcome Department of Imaging Neuroscience, London, United Kingdom), running on MATLAB vR2016a. Images were realigned using a rigid-body transformation and subsequently removed from the signal coming from non-gray matter tissue. This was performed by computing and regressing out the mean intensity coming from the CSF and white matter tissues, on a subject level. A slice timing correction and a polynomial detrending (third order) were performed prior to regressing out the 24-parameter Volterra-expanded motion parameters. Subsequently, images were normalized to the Montreal Neurological Institute standard space and smoothed with a spatial kernel of 8 mm at full width at half maximum. In order to retrieve the spontaneous synchronized oscillation of the BOLD signal during resting activity, a Butterworth band-pass filter of the fourth order was applied with frequency ranges of 0.01–0.08 Hz. Finally, we removed the first and last 5 functional volumes that could have been affected by the temporal interpolation during the band-pass filtering.

### Resting-state fMRI data analysis

To retrieve functional resting-state networks in the entire sample, we used the Multivariate Exploratory Linear Optimized Decomposition into Independent Components (MELODIC) (Beckmann and Smith 2004) implemented in the FMRIB Software Library (FSL version 6.0.1). We performed a multisubject temporal concatenation independent component analysis (ICA), with an automatic dimensionality estimation based on the latent principal components. MELODIC’s default threshold was kept (0.5), which places an equal loss on false positives and false negatives. After the estimation of spatial independent components (ICs), biologically relevant resting-state networks were selected by visual inspection and further controlled using spatial correlation against a set of 17 previously defined rsFC maps, to enable the labeling of each network (Yeo et al. 2011). Afterwards, we used the dual regression method (Nickerson et al. 2017) to regress the estimated group spatial resting-state maps against each individual’s filtered time series. This procedure generates subject-specific time-courses for each component (stage 1) along with individual images encapsulating the weights of each functional network (stage 2), which were used as input for group inferential statistics (please refer to Section 2.7). Furthermore, in order to assess group differences between networks, subject-specific time courses were analyzed using the FSLnets package (https://fsl.fmrib.ox.ac.uk/fsl/fslwiki/FSLNets), to compute the individual correlation matrices, with each matrix element being a given resting-state network.

### CSF sampling and analysis

CSF samples were obtained by lumbar puncture following previously described standard procedures (Milà-Alomà et al. 2020). Briefly, CSF was collected into a 15 mL sterile polypropylene sterile tube (Sarstedt, Nümbrecht, Germany; cat. no. 62.554.502). CSF was aliquoted in volumes of 0.5 mL into sterile polypropylene tubes (0.5 mL Screw Cap Micro Tube Conical Bottom; Sarstedt, Nümbrecht, Germany; cat. no. 72.730.005) and immediately frozen at −80 °C. Overall, the time between collection and freezing was less than 30 min. All the determinations were done in aliquots that had never been previously thawed. Aβ40 as well as Aβ42 concentrations were determined with the NeuroToolKit (Roche Diagnostics International Ltd) on cobas Elecsys e601 (Aβ42) and e411 (Aβ40) instruments at the Clinical Neurochemistry Laboratory, University of Gothenburg, Sweden. To increase sensitivity, the ratio between Aβ42 and Aβ40 was finally calculated (Lewczuk et al. 2017). Individuals with CSF Aβ42/40 higher than 0.071 were considered as Aβ negative (Milà-Alomà et al. 2020).

### Neuropsychological assessment

The neuropsychological assessment took place on average 10.6 months (range between 8.4 and 11.9 months) before the MRI session. We evaluated 4 cognitive domains, including episodic memory (EM), working memory (WM), abstract reasoning (AR), and cognitive processing speed (CPS), for which we have delineated the underlying cerebral morphology in a subsample of the present study (Cacciaglia, Molinuevo, Falcon, et al. 2018b; Cacciaglia et al. 2019). EM was assessed with the total paired recall and total free recall (TFR) scores of the Spanish adapted version of the memory binding test (Gramunt et al. 2016), an instrument that was developed for the detection of subtle EM impairments in the cognitively intact population. WM, AR, and CPS were assessed with the digit span, the similarities, and the coding subtest of the Wechsler Adult Intelligence Scale-Fourth Edition (Wechsler 2008), respectively. A description of the administration procedure is available in the online Supplementary Material.

### Statistical analyses

Due to the relatively small number of ε2/ε2 participants (*n* = 5), the groups ε2/ε2 and ε2/ε3 were collapsed into one group. *APOE* group differences in demographic and cognitive data were assessed with univariate analysis of variance or Fisher’s chi-square for continuous or categorical variables, respectively. When assessing *APOE*-related differences in cognitive performance, age, sex, and years of education were modeled as covariates. The impact of *APOE* status on rsFC was assessed by setting up a design matrix with 5 dummy regressors each coding the different *APOE* genotypes (i.e. ε2/ε2 + ε2/ε3, ε3/ε3, ε2/ε4, ε3/ε4, and ε4/ε4). Age, sex, and years of education were modeled as covariates. Contrasts of interest were performed in order to test 3 different genetic models of penetrance of the ε4 allele: additive, dominant, and recessive. Briefly, an additive model predicts an incremental response of the quantitative trait proportional to the allelic load (i.e. 0, 1, or 2 copies of the risk allele), whereas a dominant model predicts a common response to 1 or 2 copy of the risk allele (i.e. ε4-carriers vs. NC). Finally, a recessive model predicts a common response to zero or one copy of the risk allele (i.e. NC and ε4-heterozygotes [ε4HET] vs. ε4-homozygotes [ε4HMZ]) (Clarke et al. 2011). Besides the genotypic contrasts, we further modeled 2 additional contrasts considering the ε3-homozygotes (ε3HMZ) as the reference group (i.e. ε4HET vs. ε3HMZ, and ε4HMZ vs. ε3HMZ). The above defined design matrix and contrasts of interest served to test *APOE*-ε4 related differences both within and between networks. For this purpose, we used nonparametric permutations with 5,000 runs implemented in FSL (Winkler et al. 2014), using threshold-free cluster enhancement (Smith and Nichols 2009). For both the within- and between-network analyses, significance threshold was set to *P* < 0.05 implementing a family-wise error (FWE) rate correction.

To determine whether *APOE*-ε4 modulated the association between rsFC and cognitive performance, we set up a general linear model in the SPSS package (IBM Corp. Released 2012. IBM SPSS Statistics for Windows, Version 21.0. Armonk, NY: IBM Corp.), where cognitive scores served as dependent variable in each model separately, while *APOE*-ε4, rsFC estimated loadings, age, sex, as well as the interaction term between *APOE*-ε4 and rsFC, were set as independent predictors. Resulting *P*-values were subsequently corrected using a false discovery rate (FDR) approach.

## Results

### Participants’ demographics and cognitive data

Table 1 summarizes sample’s demographic information stratified across *APOE*-ε4 subgroups along with cognitive scores. NC, ε4HET, and ε4HMZ did not significantly differ in years of education, proportion of males/females, or total intracranial volume. Similarly, there were no significant differences in any cognitive variable after adjusting for covariates. However, ε4HMZ were significantly younger than both NC (post hoc *P*-value = 0.01) and ε4HET (post hoc *P*-value <0.01). For this reason, age was included as covariate in all subsequent analyses. Moreover, to validate our findings, we repeated the analyses in a subsample of study participants, which included a subset of NC and ε4HET participants, matched for age as well as other demographic variables with respect to the ε4HMZ group.

**Table 1:** Sample characteristics.

	Whole sample (*n* = 429)	NC (*n* = 205)	ε4HET (*n* = 166)	ε4HMZ (*n* = 58)	*P*-value
	*M* (SD)	*M* (SD)	*M* (SD)	*M* (SD)	
Age, years	58.27 (7.43)	58.24 (7.52)	59.45 (7.36)	55.02 (6.38)	<0.01
Sex, f/m	268/161	138/67	94/72	36/22	0.11
Education, years	13.50 (3.57)	13.58 (3.61)	13.49 (3.54)	13.28 (3.56)	0.85
TIV, mm^3^	1474.25 (145.62)	1459.39 (149.10)	1484.14 (141.62)	1498.25 (145.62)	0.11
TPR^a^	24.23 (4.47)	24.01 (4.83)	24.26 (4.21)	24.97 (3.78)	0.37
TFR^a^	16.65 (5.20)	16.63 (5.32)	16.31 (5.19)	17.72 (4.76)	0.65
WAIS DS^a^	24.71 (5.07)	24.54 (5.23)	24.55 (4.75)	25.79 (5.35)	0.32
WAIS similarities^a^	22.40 (4.70)	22.43 (4.80)	22.35 (4.59)	22.43 (4.72)	0.97
WAIS coding^a^	64.66 (15.21)	64.98 (16.03)	63.09 (14.68)	67.95 (13.23)	0.72

TIV, total intracranial volume; M, mean; SD, standard deviation; TPR, total paired recall; DS, digit span; WAIS, Wechsler Adult Intelligence Scale. ^a^Analyses corrected for age, sex, and years of education.

### ICA output in the whole sample

**Fig. 1:**
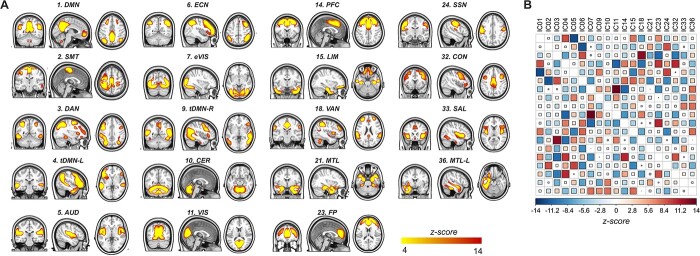
Functional resting-state networks across the entire sample. A) Nineteen biologically relevant spatial independent components identified in the whole sample, projected over single axial slices. B) *Z*-transformed connectivity matrix illustrating the spatiotemporal correlations among the identified networks. The intensity of the association between each network is encoded by both the square size and color. AUD, auditory; CER, cerebellar; CON, cingulo-opercular; DAN, dorsal attention; DMN, default mode network; ECN, executive control; eVIS, extrastriate visual; FP, frontal pole; LIM, limbic; MTL-L, left medial temporal; PFC, prefrontal; SAL, salience; SMT, somatomotor; SSN, somatosensory; tDMN-L, left temporal default mode network; tDMN-R, right temporal default mode network; TPB, temporo-basal; VAN, ventral attention; VIS, visual.

ICA retrieved 36 ICs, which were reduced to 19 biologically relevant networks after applying the selection criteria described above. Most of the retrieved spatial ICs mapped onto previously described large-scale intrinsic functional networks, such as the DMN (IC01, IC04, IC09), the executive control network (IC06), the dorsal (IC03) and the ventral (IC18) attention networks, along with sensory networks (i.e. IC02, IC05, IC07, IC11). Other ICs captured sample-specific spatiotemporal patterns, such as the left-lateralized medial temporal lobe network (IC36). Figure 1 shows the 19 selected networks overlaid over axial slices, along with the *z*-score transformed connectivity matrix, calculated across the entire group.

### Within-network results

Table 2 summarizes the main effects of *APOE*-ε4 found in our cohort.

**Table 2:** Main effects of *APOE*-ε4 within networks.

Network	Contrast	*t*-value	pFWE	Brain region	*x*	*y*	*z*
DMN (IC01)	ε4HET > ε3HMZ	4.75	<0.01	ACC	10	46	4
tDMN-L (IC04)	ε4-additive	4.12	0.02	pMTG	−50	−46	12
eVIS (IC07)	ε4-recessive	3.51	0.03	IOG	38	−86	−4
LIM (IC15)	ε4-recessive	3.43	0.04	OFC	34	62	−12
MTL-L (IC36)	ε4-additive	3.11	0.04	HC	−22	2	−16

HC, hippocampus; IOG, inferior occipital gyrus; LIM, Limbic network; MTL-L, left medial temporal network; OFC, orbitofrontal cortex; pFWE, family-wise corrected *P*-value; tDNM-L, left temporal default mode network; ε3HMZ, homozygotes for *APOE*-ε3; ε4HET, heterozygotes for *APOE*-ε4.

**Fig. 2:**
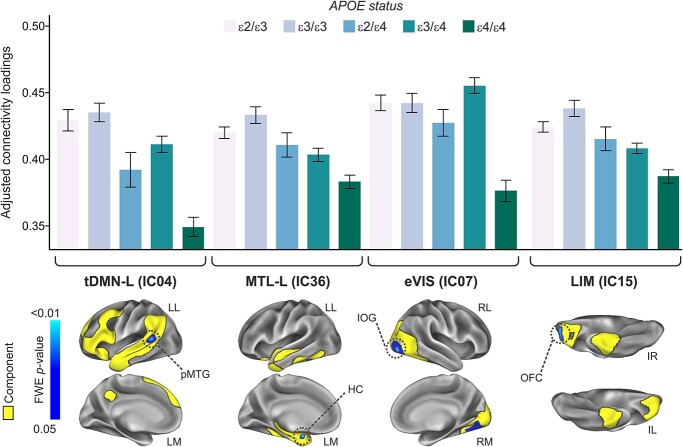
Main effects of *APOE*-ε4 on rsFC. The genotypic additive model retrieved a significant main effect of *APOE*-ε4 on the left temporal default mode (tDMN-L) and medial temporal (MTL) networks, while the recessive model yielded significant effects in the eVIS as well as the limbic (LIM) networks. LL, left lateral; LM, left medial; RL, right lateral; RM, right medial; IL, inferior left; IR, inferior right; pMTG, posterior medial temporal gyrus; HC, hippocampus; IOG, inferior occipital gyrus; OFC, orbitofrontal cortex.

**Fig. 3:**
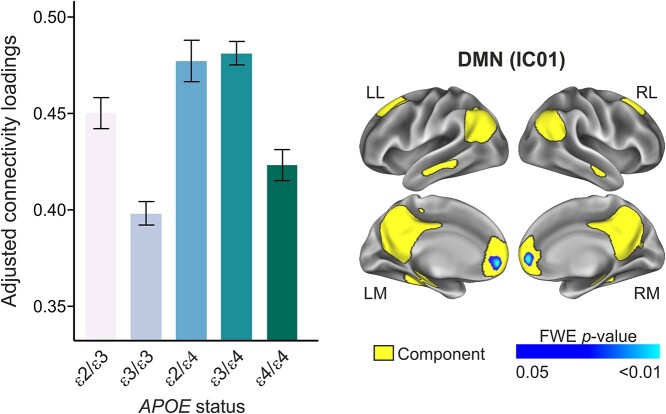
Main effect of *APOE*-ε4 on the DMN. Compared with the reference group (ε3HMZ), *APOE*-ε4 heterozygote group (ε2/ε4 + ε3/ε4) showed a higher rsFC in the DMN, mapping onto the bilateral ACC.

The additive contrast (i.e. ε4HMZ < ε4HET < NC) yielded a significantly reduced rsFC in carriers of the ε4 risk allele within 2 networks, namely, the left temporal DMN (tDMN-L, IC04) and the left medial temporal network (MTL-L, IC36). These effects mapped onto the left posterior middle temporal gyrus (pMTG) and the head of the left hippocampus, respectively (Fig. 2). These results appeared to be driven by the ε4HMZ group, as we observed a significantly reduced rsFC in those 2 networks also when testing the ε4HMZ < ε3HMZ contrast. The genotypic recessive model (i.e. ε4HMZ < rest of the subjects) yielded a significantly reduced rsFC within 2 networks, namely the extrastriate visual network (eVIS, IC07) and the limbic network (LIM, IC15). These effects mapped onto the right inferior occipital gyrus and the right orbitofrontal cortex, respectively (Fig. 2). Finally, there was a significantly increased connectivity in ε4HET (i.e. *APOE*-ε2/ε4 + *APOE*-ε3/ε4) compared with *APOE*-ε3/ε3 within the DMN (IC01), in the anterior cingulate cortex (ACC) (Fig. 3). A nominal significant effect was also observed within the DMN in the ACC, when inspecting the ε4HMZ > ε3HMZ contrast, which however did not survive correction for multiple testing (pFWE = 0.07, data not shown).

### Between-network analysis

**Fig. 4:**
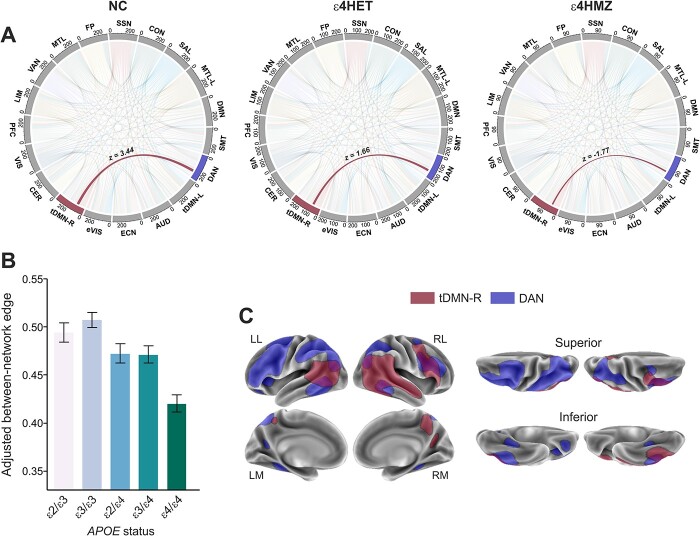
Between-networks effect of *APOE*-ε4. Network modeling analysis retrieved a significant main effect of *APOE*-ε4 under a genotypic additive assumption, between the right temporal default mode (tDMN-R) and the DAN networks. Specifically, *APOE*-ε4 carriers showed a reduced rsFC between these 2 networks.

The additive model yielded a significantly reduced rsFC in *APOE*-ε4 carriers between the dorsal attention network (DAN, IC03) and the right temporal DMN (tDMN-R, IC09) (β = 0.81; *t* = 3.07; *P* = 0.002; pFWE = 0.03), indicating a diminished connectivity between these 2 brain networks as a function of the ε4 allelic load (Fig. 4). No significant differences were found between any other networks in any contrast.

### Associations with incipient neurodegeneration and cognitive data

**Fig. 5:**
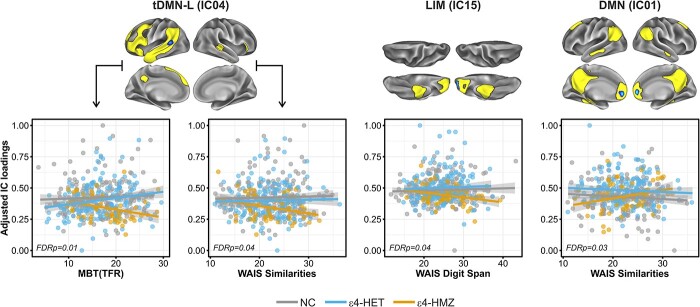
*APOE*-ε4 modified the associations between rsFC and cognitive performance. Significant interactions between *APOE*-ε4 status and cognitive performance in different domains indicated that ε4HMZ group exhibits a distinctive pattern of linear association between rsFC in the areas of significant and performance in different cognitive domains. MBT (TFR), memory binding test (total free recall); WAIS, Wechsler Adult Intelligence Scale.

To determine the functional implication of the rsFC differences we found among the *APOE* subgroups, we assessed the interactions between connectivity parameters and measures of cognitive performance in multiple domains. For the memory domain, we found that *APOE*-ε4 status modified the association between TFR and rsFC in the tDMN-L (IC04) (*F*_2,426_ = 4.72, *P* = 0.009, pFDR = 0.036). In non-memory domains, we found significant interactions between *APOE*-ε4 and AR in the tDMN-L (IC04) (*F*_2,426_ = 3.89, *P* = 0.021, pFDR = 0.042), as well as the DMN (IC01) (*F*_2,426_ = 7.029, *P* = 0.008, pFDR = 0.032). Finally, *APOE*-ε4 modified the association between rsFC in the limbic network (IC15) and WM (*F*_1,428_ = 6.487, *P* = 0.011, pFDR = 0.044) (Fig. 5). No significant interactions were found between *APOE*-ε4 status and between-network connectivity in driving cognitive performance, in any domain.

To aid the interpretation of these interactions, we performed a post hoc analysis aimed to identify putative differences in gray matter volume (GMv) among the different *APOE* subgroups and their associations with cognitive performance. To this aim, we performed a voxel-based morphometry modeling the effects of the 5 *APOE* genotype groups along with sex, age, and years of education as covariates. Please refer to the Supplementary Materials for a detailed description of the structural MRI data preprocessing. We found that, compared with NC and ε4HET, ε4HMZ displayed significant GMv reductions in the right posterior hippocampus (Supplementary Fig. S1A). Furthermore, hippocampal GMv was negatively associated with rsFC in the tDMN-L (IC04), in the ε4HMZ but not in the rest of the participants (Supplementary Fig. S1B).

### Complementary analysis in CSF Aβ-negative individuals

To determine to what extent the observed effects were specific to *APOE* genetic variance and independent on brain amyloidosis, we reanalyzed data from a subsample of individuals who were categorized as Aβ-negative, based on their levels of CSF Aβ42/40 ratio in a retrospective analysis_._ While such an approach is blind to the actual Aβ values when MRI data were acquired, it allows to identify those who did not have Aβ pathology at that time. Of the entire sample (*n* = 429), CSF was available for 91 individuals. Of those, 54 were Aβ-negative (see Supplementary Table S1 for a sample description). Supplementary Table S2 shows main effects of *APOE*-ε4 on this subsample. We confirmed a significant *APOE*-ε4 dose-dependent reduced connectivity in the eVIS (IC07), with this effect mapping onto the right inferior occipital gyrus. Moreover, we found a reduced connectivity within the left MTL network (IC36) for the contrasts ε4HMZ < ε3HMZ, as well as the ε4HET < ε3HMZ, with these effects being significant in the left inferior temporal gyrus and the left temporal pole, respectively. There was no significant difference between networks as a function of *APOE*-ε4 status in between-network connectivity, in the Aβ negative subsample. Finally, none of the interactions between *APOE*-ε4 and cognitive performance found in the entire sample was significant.

### Analyses conducted in age-matched APOE subgroups

To further control that the participants’ age difference across *APOE* genotype subgroups did not influence our main findings, we repeated key analyses in a subsample of individuals including the ε4HMZ subgroup along with NC and ε4HET individuals, matched for sample size, age, sex, and years of education (Supplementary Table S3). To this aim, we computed the pairwise distance for these demographic variables between the NC and ε4HMZ and between the ε4HET and ε4HMZ subgroups, using the pdist2 function in Matlab (https://es.mathworks.com/help/stats/pdist2.html), selecting default distance parameters (i.e. Euclidean distance). In the resulting subsample, we could replicate each of the key findings obtained in the entire sample, although the increased rsFC in the anterior DMN previously observed for the contrast ε4HET > *APOE*-ε3/ε3, only reached a statistical trend (Supplementary Fig. S2A and B). Finally, we could replicate the results from the entire sample on the interactions between *APOE* status and cognitive performance (Supplementary Fig. S2C).

## Discussion

The present work aimed to characterize the impact of *APOE*-ε4 risk variant for AD on the functional organization of the brain, as reflected by resting-state networks, in cognitively intact middle-aged individuals. We capitalized on our cohort harboring a significantly higher number of *APOE*-ε4 homozygotes than previously reported in single-site studies of healthy subjects. This unique sample characteristic enabled testing different models of genetic penetrance, which are informative on the mechanisms underlying the enhanced AD prevalence in *APOE*-ε4 carriers. Moreover, we inspected whether *APOE*-ε4 further modulated the association between rsFC and cognitive performance in different domains.

We reported significant effects of *APOE*-ε4 both within- and between-networks and further validated key findings in a subsample of individuals, which were matched for age across the *APOE* genotype categories.

First, we found a reduced rsFC within the tDMN-R (IC04) and MTL-L (IC36), in carriers of the ε4 allele, under a genotypic additive model assumption (i.e. ε4HMZ < ε4HET < NC). These effects mapped onto the left posterior MTG and left anterior hippocampus, respectively. The recessive contrast, which identifies statistical effects uniquely driven by the homozygote group, retrieved a reduced rsFC within the eVIS (IC07) and LIM (IC15) networks, respectively, mapping onto the right inferior occipital gyrus and left orbitofrontal cortex. Furthermore, we observed that, compared with NC, ε4HET displayed an increased rsFC within the DMN, mapping onto the ACC. The ε4HMZ group also displayed a higher anterior DMN connectivity compared to NC, although on a nominal significance level. Our finding is in line with previous studies documenting an increased rsFC in the anterior DMN in asymptomatic ε4-carriers individuals (Filippini et al. 2009; Fleisher et al. 2009). Our interaction data indicate that *APOE*-ε4 further modulated the association between rsFC and cognitive performance in multiple domains. Specifically, we report that, in ε4HMZ but not in the rest of participants, better AR was related to a higher anterior DMN rsFC, but to a reduced rsFC in the tDMN-L. Again, only in ε4HMZ, better EM and WM were negatively related to rsFC in the tDMN-L and LIM networks, respectively. Together, these interactions suggest that genotypic group differences in rsFC may underlie a compensatory response possibly in the face of incipient AD-related pathology, aiding an efficient cognitive performance. Our structural MRI data support this interpretation, given that ε4HMZ displayed a reduced posterior hippocampal volume, which was negatively associated with connectivity estimates in the tDMN-L. In a similar vein, others have reported that in CU individuals, *APOE*-ε4 carriers showed a reversed association, compared with NC, between rsFC and cognitive performance supporting the compensation account (Westlye et al. 2011; Shafer et al. 2021). Alternatively, our interaction data may represent a compensatory readjustment due to incipient Aβ deposition. In fact, at the mean age in our sample, about 50% of ε4 homozygotes are expected to harbor Aβ pathology (Jansen et al. 2015). Moreover, it should be noted that we could not replicate any of the observed interactions between *APOE*-ε4 and cognitive performance in the Aβ-negative subsample. However, the lack of Aβ biomarkers for the entire sample prevents us from drawing a more robust conclusion.

With respect to the posterior subdivision of the DMN, previous studies have reported a functional disconnection in *APOE*-ε4 carriers compared to NC, targeting the precuneus (Sheline et al. 2010; Machulda et al. 2011; Damoiseaux, Seeley, et al. 2012b). We found no evidence for a reduced connectivity within the posterior DMN in our sample. Such a discrepancy may be related to the difference in the mean age of the participants (Chen et al. 2017; Klaassens et al. 2017). In fact, in 2 of the previous studies (Machulda et al. 2011; Damoiseaux, Seeley, et al. 2012b), the sample was on average older than ours, possibly reflecting a more advanced disease stage, with a possible co-occurrence of abnormal Aβ or tau. On the other hand, the study of Sheline et al. (2010) adopted a seed-based connectivity approach on the precuneus, whereas our methodology based on ICA was spatially unbiased. Yet, our finding of a reduced rsFC in the tDMN-L highlights a diminished functional organization of the temporal subdivision of the in *APOE*-ε4 carriers in a gene dose-dependent manner, which has not been reported in previous studies so far. Similarly, Turney et al. (2020) reported a decreased rsFC in the temporal DMN subsystem in asymptomatic ε4 carriers compared to NC, without, however, testing different models of genetic penetrance. The tDMN-L (IC04) included the left pMTG, as well as lateral temporal and inferior frontal gyrus, all areas that are implicated in semantic control and retrieval (Snijders et al. 2010; Noonan et al. 2013). In particular, the left pMTG is a functional nexus drawing together 2 well-documented large-scale networks, namely the DMN and the ECN, which are involved in automatic semantic processing and executive control, and thus allowing a highly specialized patterns of retrieval (Davey et al. 2016). Again, our interaction data suggest that, in middle-aged CU individuals, connectivity reduction of the left pMTG within the tDMN-L network supports better episodic recall as well as AR, in the ε4HMZ group but not in the rest for the subjects. Altogether, these findings suggest that the ε4HMZ group, which presents the highest risk for developing AD in the future (Corder et al. 1993; Farrer et al. 1997), relies on a distinctive cerebral functional network to achieve a normative cognitive performance. This may have the cost of a network failure later in time, as suggested previously (Jones et al. 2016). In this vein, our current data are consistent with our previous results showing that *APOE*-ε4 modifies the linear association between gray matter volumes in different brain regions and cognitive performance (Cacciaglia et al. 2019). It should also be noted that the pMTG, together with frontal and parietal areas, has been reported as a key region of hypometabolism in CU individuals, as a function of *APOE*-ε4 allelic load (Reiman et al. 2005; Protas et al. 2013), and also in AD patients compared with control subjects (Mosconi et al. 2009; Landau et al. 2011). Therefore, our data extend those earlier findings showing a concomitant rsFC reduction in this area, in a dose-dependent manner.

The genotypic additive model also retrieved a reduced rsFC within the MTL-L network, mapping onto the anterior hippocampus. Typically, hippocampal connectivity as a function of *APOE*-ε4 in CU individuals has been studied in relation to the DMN, with a number of studies reporting increased rsFC between the anterior hippocampus and frontal as well as parietal regions (Filippini et al. 2009; Westlye et al. 2011; Trachtenberg et al. 2012). However, no study to our knowledge has reported a decreased rsFC of the hippocampus within the MTL network. Hence, our result may represent a counterpart of previously reported data, suggesting that a higher connectivity between the hippocampus and DMN regions occurs at the cost of a less efficient functional integration of the hippocampus with the MTL network. Finally, we found a decreased rsFC in ε4HMZ compared to the rest of study participants, in the eVIS. Regions belonging to the eVIS regulate visual attention and show robust functional co-activation during encoding of new information, with hippocampal (Diersch et al. 2021) and prefrontal cortices (Grady et al. 2003). Moreover, eVIS areas show progressive disconnection with the DMN in aging (Chhatwal et al. 2018) and neurodegeneration not due to AD (Rektorova et al. 2012). Interestingly, we could replicate this finding also in the subsample with negative markers of Aβ, suggesting a neurodevelopmental effect of *APOE*-ε4 on this network, which may be a neural signature not related to the presence of AD pathology. Further research is needed to track the connectivity of the visual system in preclinical AD.

Lastly, in our between-network analysis, we observed a reduced rsFC between the DAN (IC03) and the tDMN-R (IC09) in a gene dose-dependent manner. Interestingly, one study found an increased negative correlation in patients with an incipient dementia due to AD, between functional networks that normally display an intrinsic anticorrelation (Wang et al. 2007). In other words, the authors found that regions belonging to “task-positive” and “task-negative” networks (Fox et al. 2005), were further anticorrelated in AD patients compared with controls. Thus, our finding of a reduced functional coupling between the temporal subdivision of the DMN and the DAN is reminiscent of a functional connectivity pattern observed in early AD and may underlie a network correlate of the increased AD risk along with the number of ε4 alleles. Moreover, a diminished rsFC between 2 major cognitive networks, such as the salience network and the ECN, was previously observed cross-sectionally in ε4 carries, which is in line with our finding of an increased segregation between 2 networks critical for an efficient cognitive processing in carriers of the risk allele, at least in the asymptomatic stages (Ng et al. 2018). The lack of core AD biomarkers for the entire sample, as well as the unavailability of follow-up data, represents 2 limitations of the present work. Future studies shall investigate the contribution of risk genes, Aβ and tau pathologies separately, to disentangle their specific impact on the functional connectome, in longitudinal designs.

In summary, in CU individuals, we report different genotypic effects of *APOE*-ε4 both within and between networks, in large-scale systems related to cognitive (tDMN-L, MTL-L) as well as more sensory (eVIS) processing. Some of these effects were replicated in Aβ-negative individuals, and therefore may lie upstream AD pathology. Such a network reorganization predicted better cognitive performance in the high-risk ε4HMZ group, which concomitantly displayed a reduced hippocampal volume compared with the rest of the sample, thus supporting the idea of a network-based functional compensation.

## Supplementary Material

Supplementary_Materials_RCacciaglia_Cer_Cortex_Revision_bhac239
