# Supplementary material for: Genotypic effects of APOE-ε4 on resting-state connectivity in cognitively intact individuals support functional brain compensation
Source: Cereb Cortex. 2022 Jun 27;33(6):2748–60. doi: 10.1093/cercor/bhac239 (PMC10016049; doi:10.1093/cercor/bhac239)
Supplement: Supplementary_Materials_RCacciaglia_Cer_Cortex_Revision_bhac239 [file supplementary_materials_rcacciaglia_cer_cortex_revision_bhac239.docx]

**Title:** Genotypic effects of *APOE*-ε4 on resting-state connectivity in cognitively intact individuals support functional brain compensation

**Authors:** Raffaele Cacciaglia, Grégory Operto, Carles Falcón, José Maria González de Echavarri-Gómez, Gonzalo Sánchez-Benavides, Anna Brugulat-Serrat, Marta Milà-Alomà, Gwendlyn Kollmorgen, Ivonne Suridjan, Kaj Blennow, Henrik Zetterberg, José Luis Molinuevo, Marc Suárez-Calvet and Juan Domingo Gispert, for the ALFA study.

**Supplementary Materials**

**Structural MRI data preprocessing and analysis**

Gray matter (GM) was segmented from T1 images using the new segment function implemented in Statistical Parametrical Mapping software (SPM 12, Wellcome

Department of Imaging Neuroscience, London, UK), and located into a common space for subsequent normalization, using a 12-affine parameter transformation. Segmented GM images were used to generate a reference template of the sample, which was warped into a standard Montreal Neurological Institute (MNI) space using the high dimensional

DARTEL toolbox (Ashburner, 2007). The generated flow fields and normalization parameters were then implemented to normalize the native GM images to the MNI space. In order to preserve the native local amount of GM volume, we applied a modulation step, where each voxel signal's intensity was multiplied by the Jacobian determinants derived

from the normalization procedure (Good et al., 2001). Quality control of normalization was assured by checking the sample homogeneity with the computational anatomy toolbox (CAT12) (<http://dbm.neuro.unijena.de/cat/>) using non-smoothed data, which did not return errors in the registration procedure in any subject. Finally, images were spatially smoothed with a 6mm full-width at half maximum (FWHM) Gaussian kernel. Total intracranial volume (TIV) was computed by summing the segmented GM, white matter (WM) and cerebrospinal fluid (CSF) volumes for each participant. Group analysis was conducted with the Statistical Parametric Mapping software (SPM12, Wellcome Department of Imaging Neuroscience, London, UK). To determine the impact of *APOE*-ε4 gray matter volume, we set up a general linear model (GLM) including five dummy regressors each coding the ε2/ε3, ε2/ε4, ε3/ε3, ε3/ε4, and ε4/ε4 genotype groups as predictors, as well as age, sex, years of education and TIV as covariates. Next, different t-contrasts were performed to test the distinct models of genetic penetrance, namely the dominant, recessive and additive effects, as proposed for the analysis of quantitative trait loci (Clarke et al., 2011). The resulting parametric maps were masked with a binary region of interest encompassing the bilateral hippocampus. Results were considered significant if surviving a whole brain voxel-wise statistical threshold of *p* < .005 applying a cluster extent threshold correction of 50 voxels.

**Procedures for neuropsychological assessment**

Episodic memory was assessed using the Memory Binding Test (MBT). Previous studies have established the ability of the MBT (formerly referred as Memory Capacity Test [MCT]) to discriminate subjects with cerebral Aβ deposition (Papp et al., 2015), to successfully discriminate individuals with mild cognitive impairment (MCI) from normal elderly subjects (Buschke et al., 2017) and also successfully predict the incidence of MCI longitudinally (Mowrey et al., 2016). The MBT uses a controlled learning procedure to ensure that any recall deficit can be attributed to effective memory impairment and not to deficiency in any other cognitive strategies. During administration, the examinee sequentially learns two lists of 16 words written in cards, where each card contains four words. The lists share semantic categories, which are used both to control the encoding of the words in learning and as cues during cued recall trials. In the present study, we included the two main MBT outcome, namely Total Paired Recall (TPR) and Total Free Recall (TFR). TPR indexes the immediate recall of both lists after semantic cueing, while TFR provides a measure of the immediate free recall, with no sematic cueing, of both lists.

Cognitive processing speed was assessed with the Coding subtest of the Wechsler Adult Intelligence Scale-Fourth Edition (Wechsler, 2012). Here, participants are given keys that match numeric digits spanning from 1 to 9 with a symbol. The task is to write down the correct symbol next to a list of numbers as quickly as possible. Finally, working memory (WM), abstract reasoning (AR) were assessed with the digit span and the Similarities subscales of the WAIS. For WM assessment, we considered the sum of the digit span forward, backward, and sequencing (digit-span total).

**Analyses in a reduced sample matched for age**

*Within network analyses*

In the age-matched subsample, we found a significantly reduced rsFC as a function of the *APOE*-ε4 allelic load (*e.g.,* additive contrast) in the tDMN-L (pFWE = 0.04) and in the LIM (pFWE = 0.02) networks. A significant recessive effects was found in the MTL-L (pFWE =0.02) and the eVIS (pFWE = 0.02) networks. Finally, a statistical trend indicated a in increased rsFC in the e4-HET group compared with the *APOE*-ε3/ε3, in the anterior DMN (pFWE =0.07) (**Fig. S2a**).

*Between-network analyses*

As in the entire sample, in the reduced subsample, *APOE*-ε4 was associated with a reduced connectivity between the ECN-L (IC03) and the tDMN-R (IC09), when testing the additive model (p=0.032) (**Fig. S2b**).

*Associations with incipient neurodegeneration and cognitive data*

*APOE*-ε4 homozygotes displayed significantly different linear associations between rsFC and multiple measures of cognitive performance (**Fig. S2c**). Moreover, as observed in the entire dataset, we found that, compared with NC and ε4HET groups, ε4HMZ displayed a significantly reduced right posterior hippocampal volume (t_167_ = 3.37, p<0.001, cluster size [k] = 107, x = 17, y = -42, z = -3)

**Table S1 – Characteristics within the CSF Aβ42/40 negative subsample**

|  |  | **NC ( n=29)** |  | **ε4HET (n=18)** |  | **ε4HMZ (n=7)** |  | **P-value** |
| --- | --- | --- | --- | --- | --- | --- | --- | --- |
|  |  | ***M (SD)*** |  | ***M (SD)*** |  | ***M (SD)*** |  |  |
| **Age, y** |  | 57.97 (4.85) |  | 59.33 (3.51) |  | 52.72 (5.05) |  | <0.01 |
| **Sex, f/m** |  | 18/11 |  | 7/11 |  | 5/2 |  | 0.19 |
| **Education, y** |  | 12.00 (3.49) |  | 14.28 (3.41) |  | 13.00 (3.46) |  | 0.17 |
| **TIV, mm^3^** |  | 1460.94 (119.21) |  | 1522.87 (151.21) |  | 1456.10 (203.33) |  | 0.93 |
| **TPR*** |  | 23.41 (4.74) |  | 25.73 (2.88) |  | 22.29 (4.68) |  | 0.15 |
| **TFR*** |  | 16.24 (4.38) |  | 18.61 (3.41) |  | 14.29 (3.45) |  | 0.02 |
| **DS*** |  | 25.68 (5.81) |  | 25.38 (4.91) |  | 23.85 (6.33) |  | 0.22 |
| **Similarities** |  | 22.41 (4.68) |  | 23.00 (3.91) |  | 19.42 (4.57) |  | 0.11 |
| **Coding*** |  | 65.93 (12.99) |  | 64.44 (13.40) |  | 68.71 (15.97) |  | 0.54 |

NC: Non-carriers; ε4HET: ε4-heterozygotes; ε4HET: ε4-homozygotes; TIV: total intracranial volume; M: mean; SD: standard deviation; TPR: Total paired recall; TFR: Total free recall; DS: Digit Span; *Analyses corrected for age, sex and years of education

**Table S2 – Main effects of *APOE*-ε4 within networks in a subsample with negative CSF Aβ markers**

| Networks | **Model** | **t-value** | **pFWE** | **brain región** | **x** | **y** | **y** |
| --- | --- | --- | --- | --- | --- | --- | --- |
|  |  |  |  |  |  |  |  |
| **eVIS (IC07)** | ε4-additive | 4.12 | <0.01 | Inferior Occipital | 30 | -82 | -16 |
| **MTL-L (IC36)** | ε4HMZ<ε3HMZ | 3.41 | 0.04 | Inferior Temporal | -42 | -30 | -20 |
| **MTL-L (IC36)** | ε4HET<ε3HMZ | 4.18 | <0.01 | Temporal Pole | -54 | 6 | -12 |

CSF: Cerebrospinal fluid; Aβ: beta-amyloid; eVIS: extrastriate visual network; MTL-L: Left middle temporal network;

ε4HMZ: Homozygotes for *APOE*-ε4; ε4HET: Heterozygotes for *APOE*-ε4; ε3HMZ: Homozygotes for *APOE*-ε3;

pFWE: family-wise error rate corrected p-value

**Table S3 – Characteristics within the reduced subsample matched for age**

|  |  | **NC ( n=58)** |  | **ε4HET (n=58)** |  | **ε4HMZ (n=58)** |  | **P-value** |
| --- | --- | --- | --- | --- | --- | --- | --- | --- |
|  |  | ***M (SD)*** |  | ***M (SD)*** |  | ***M (SD)*** |  |  |
| **Age, y** |  | 56.90 (7.61) |  | 56.93 (6.97) |  | 55.02 (7.01) |  | 0.24 |
| **Sex, f/m** |  | 36/22 |  | 34/24 |  | 36/22 |  | 0.89 |
| **Education, y** |  | 13.64 (3.61) |  | 13.38 (3.49) |  | 13.28 (3.56) |  | 0.85 |
| **TIV, mm^3^** |  | 1,467.91 (135.4) |  | 1,474.05 (137.75) |  | 1,498.43 (141.19) |  | 0.45 |
| **TPR*** |  | 24.41 (4.71) |  | 24.01 (5.40) |  | 24.46 (4.67) |  | 0.58 |
| **TFR*** |  | 16.86 (4.74) |  | 17.19 (5.39) |  | 17.72 (4.76) |  | 0.68 |
| **DS*** |  | 24.64 (5.37) |  | 24.50 (4.73) |  | 25.79 (5.35) |  | 0.29 |
| **Similarities** |  | 23.07 (5.07) |  | 22.05 (4.60) |  | 22.43 (4.79) |  | 0.55 |
| **Coding*** |  | 63.94 (15.41) |  | 65.49 (15.23) |  | 67.94 (13.23) |  | 0.45 |

NC: Non-carriers; ε4HET: ε4-heterozygotes; ε4HET: ε4-homozygotes; TIV: total intracranial volume; M: mean; SD: standard deviation; TPR: Total paired recall; TFR: Total free recall; DS: Digit Span; *Analyses corrected for age, sex and years of education


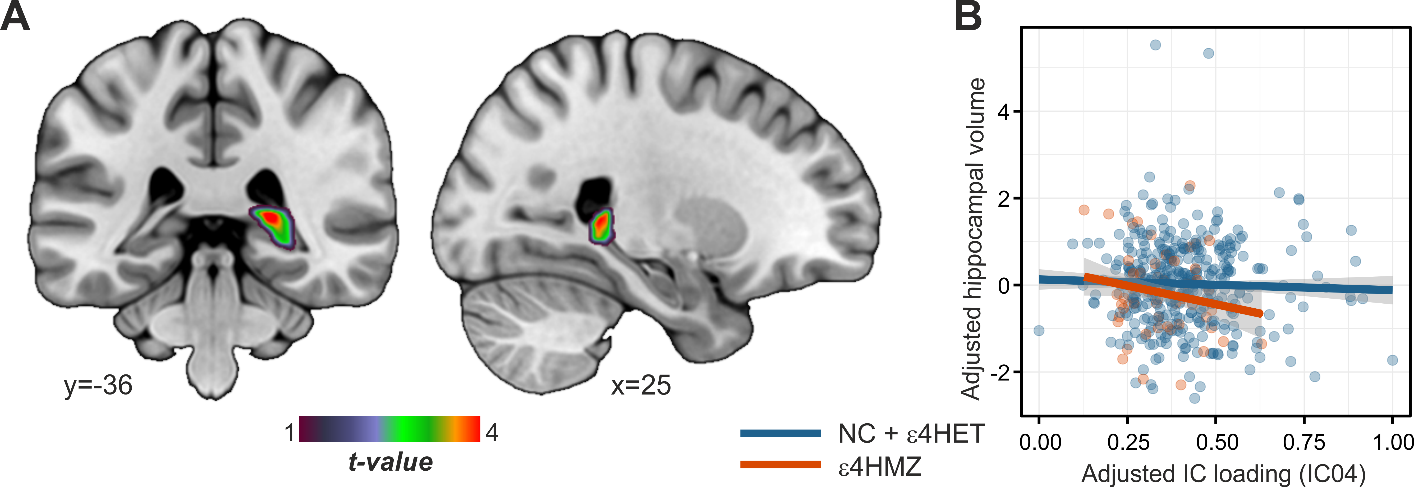


**Fig. S1 –** **A)** Compared with Non-Carriers (NC) and ε4-heterozygotes (ε4HET), e4-homozygotes (ε4HMZ) showed a reduced gray matter volume in the right posterior hippocampus. **B)** Unlike the rest of participants, ε4HMZ showed a trend to a negative association between the right posterior hippocampal volume and the connectivity loadings in the temporal subdivision of the default-mod network (tDMN-L) (IC04)


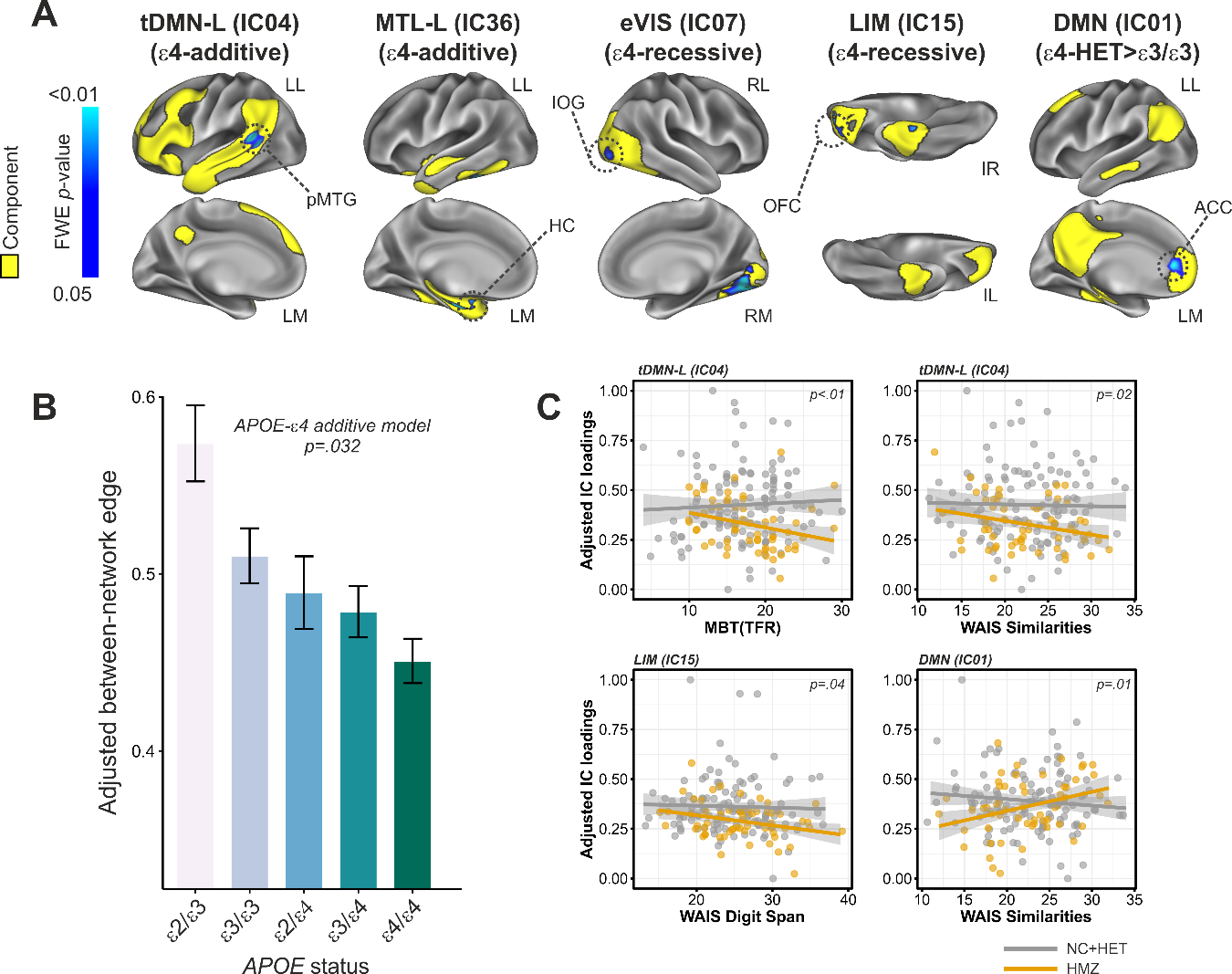


**Fig S2** – **Analyses conducted in a subsample of n=174 individuals matched for age, sex and years of education across the *APOE*-ε4 grouping factor.** **A)** Within network analysis: In the age-matched subsample, we found a significantly reduced rsFC as a function of the *APOE*-ε4 allelic load (*e.g.,* additive contrast) in the tDMN-L (pFWE = 0.04) and in the LIM (pFWE = 0.02) networks. A significant recessive effects was found in the MTL-L (pFWE =0.02) and the eVIS (pFWE = 0.02) networks. Finally, a statistical trend indicated a in increased rsFC in the ε4-HET group compared with the *APOE*-ε3/ε3, in the anterior DMN (pFWE =0.07). **B)** Between –network analysis: *APOE*-ε4 was associated with a reduced connectivity between the DAN (IC03) and the tDMN-R (IC09), when testing the additive model (p=0.032). **C)** *APOE*-ε4 homozygotes displayed significantly different linear associations between rsFC and multiple measures of cognitive performance. NC: Non-carriers; HET: ε4-heterozygotes; HMZ: ε4-homozygotes

**REFERENCES**

Ashburner, J., 2007. A fast diffeomorphic image registration algorithm. Neuroimage 38, 95-113.

Buschke, H., Mowrey, W.B., Ramratan, W.S., Zimmerman, M.E., Loewenstein, D.A., Katz, M.J., Lipton, R.B., 2017. Memory Binding Test Distinguishes Amnestic Mild Cognitive Impairment and Dementia from Cognitively Normal Elderly. Arch Clin Neuropsychol 32, 1037-1038.

Clarke, G.M., Anderson, C.A., Pettersson, F.H., Cardon, L.R., Morris, A.P., Zondervan, K.T., 2011. Basic statistical analysis in genetic case-control studies. Nat Protoc 6, 121-133.

Good, C.D., Johnsrude, I.S., Ashburner, J., Henson, R.N., Friston, K.J., Frackowiak, R.S., 2001. A voxel-based morphometric study of ageing in 465 normal adult human brains. Neuroimage 14, 21-36.

Papp, K.V., Amariglio, R.E., Mormino, E.C., Hedden, T., Dekhytar, M., Johnson, K.A., Sperling, R.A., Rentz, D.M., 2015. Free and cued memory in relation to biomarker-defined abnormalities in clinically normal older adults and those at risk for Alzheimer's disease. Neuropsychologia 73, 169-175.

Mowrey, W.B., Lipton, R.B., Katz, M.J., Ramratan, W.S., Loewenstein, D.A., Zimmerman, M.E., Buschke, H., 2017. Memory Binding Test Predicts Incident Amnestic Mild Cognitive Impairment. J Alzheimers Dis 58, 951-952.

Wechsler D, 2008. Wechsler Adult Intelligence Scale–Revised. Wechsler Adult Intelligence Scale (WAIS-IV). San Antonio, TX Psychol. Corp. 1981.
